# Autoantibodies-Abzymes with Phosphatase Activity in Experimental Autoimmune Encephalomyelitis Mice

**DOI:** 10.3390/molecules29061382

**Published:** 2024-03-20

**Authors:** Andrey E. Urusov, Kseniya S. Aulova, Georgy A. Nevinsky

**Affiliations:** 1 Institute of Chemical Biology and Fundamental Medicine, SB of the Russian Academy of Sciences, Lavrentiev Ave., 8, 630090 Novosibirsk, Russia; urusow.andrew@yandex.ru (A.E.U.);; 2 Faculty of Natural Sciences, Novosibirsk State University, 630090 Novosibirsk, Russia

**Keywords:** experimental autoimmune encephalomyelitis (EAE) model, C57BL/6 mice, catalytic antibodies, phosphatase activity

## Abstract

The exact mechanisms of MS (multiple sclerosis) evolution are still unknown. However, the development of EAE (experimental autoimmune encephalomyelitis simulating human MS) in C57BL/6 mice occurs due to the violation of bone marrow hematopoietic stem cell differentiation profiles, leading to the production of toxic for human autoantibody splitting MBP (myelin basic protein), MOG (mouse oligodendrocyte glycoprotein), five histones, DNA, and RNA. Here, we first analyzed the changes in the relative phosphatase activity of IgGs from C57BL/6 mice blood over time, corresponding to three stages of EAE: onset, acute, and remission. Antibodies have been shown to catalyze the hydrolysis of *p*-nitrophenyl phosphate at several optimal pH values, mainly in the range of 6.5–7.0 and 8.5–9.5. During the spontaneous development of EAE, the most optimal value is pH 6.5. At 50 days after the birth of mice, the phosphatase activity of IgGs at pH 8.8 is 1.6-fold higher than at pH 6.5. During spontaneous development of EAE from 50 to 100 days, an increase in phosphatase activity is observed at pH 6.5 but a decrease at pH 8.8. After mice were immunized with DNA–histone complex by 20 and 60 days, phosphatase activity increased respectively by 65.3 and 109.5 fold (pH 6.5) and 128.4 and 233.6 fold (pH 8.8). Treatment of mice with MOG at the acute phase of EAE development (20 days) leads to a maximal increase in the phosphatase activity of 117.6 fold (pH 6.5) and 494.7 fold (pH 8.8). The acceleration of EAE development after mice treatment with MOG and DNA–histone complex results in increased production of lymphocytes synthesizing antibodies with phosphatase activity. All data show that IgG phosphatase activity could be essential in EAE pathogenesis.

## 1. Introduction

Various antibodies with catalytic activities—abzymes (Abzs) against chemically stable analogs of transition states of some chemical reactions catalyzing more than 190 various reactions—are obtained as novel important enzymes (for review, see [1,2,3,4,5,6]). Natural catalytic auto-Abz splitting polysaccharides, nucleic acids, nucleotides, different oligopeptides, and proteins were detected in the blood of patients with many autoimmune diseases (AIDs) (for review, see [6,7,8,9,10,11,12,13,14,15,16,17,18,19]). Multiple sclerosis (MS) is a chronic demyelinating central nervous system pathology [20,21,22,23,24,25,26,27]. MS etiology is still unclear and the widely used theory of its pathogenesis assumes a leading role of inflammation associated with specific autoimmune reactions with myelin destruction [20,21,22,23,24,25,26,27].

It is believed that activated CD4+ myelin-reactive T cells are major mediators of MS [20,21,22,23,24,25,26,27]. Several findings imply that B cells also play an important role. Current evidence from autoimmune animal models and clinical studies show that a crucial role in MS immunopathogenesis may play auto-Abs against myelin autoantigens, which are involved in antibody-mediated demyelination [26], and oligodendrocyte progenitor cell protein, which could block remyelination by eliminating or impeding these cells [26,27]. Several studies showed that autoantibodies to myelin components and other antigens, including antibodies with catalytic activities, may be essential in the pathogenesis of MS (for a review, see [18,19]). However, the possible spectrum of such antibodies and abzymes, which could be important for developing MS, has yet to be discovered.

Several experimental autoimmune encephalomyelitis (EAE) mice models exist that mimic particular features of human MS (for a review, see [28,29,30,31,32,33,34]). As in patients with MS, mice prone to the development of EAE exhibit some indicators of MS pathology, including different typical clinical indexes, neurological, and histological evidence of pathology, including optic neuritis, plaque formation in the brain, and paralysis of some organs and the lower extremities [28,29,30,31,32,33,34]. One of these models is C57BL/6 mice. EAE evolution in C57BL/6 mice proceeds spontaneously and has a chronic-progressive course.

Some autoimmune diseases were first supposed to be a consequence of defects of hematopoietic stem cells (HSCs) [35]. Defects of HSCs were revealed in some diseases and aging [36,37,38].

Spontaneous and accelerated systemic lupus erythematosus (SLE)-prone mice (MRL-lpr/lpr) by immunization with DNA [39] and EAE-prone C57BL/6 mice with several antigens [40,41,42] are conditioned by a specific reorganization of bone marrow hematopoietic stem cells. In AIDs including EAE mice, immune-system-contravention defects were bound with distinct changes in the bone marrow profile of HSC differentiation, which is associated with the production of particular catalytic Abs-abzymes hydrolyzing nucleic acids, histones, peptides, proteins, and polysaccharides [18,19,39,40,41,42]. With some exceptions, abzymes with DNase, RNase, and proteolytic catalytic activities are absent in the blood of conditionally healthy volunteers, or they usually have shallow activities [18,19,39,40,41,42]. Auto-abzymes with several enzymatic activities were revealed as the first statistically significant and earliest markers of many AIDs beginning and following development [18,19,39,40,41,42],

DNase and histone-hydrolyzing Abzs of SLE [43] and multiple sclerosis patients [18,19] are detrimental and cytotoxic because they induce cell apoptosis, which accelerates the development of AIDs. Abzs against myelin basic protein (MBP) and myelin oligodendrocyte glycoprotein (MOG) possessing proteolytic activity in MS and SLE patients may attack specific proteins of the axon myelin-proteolipid sheath. Consequently, these auto-abzymes could also play a detrimental role in MS pathogenesis.

EAE C57BL/6 mice were used recently to study the possible mechanisms of spontaneous, DNA–protein complexes and MOG-accelerated development of EAE [18,19,40,41,42,43]. It was demonstrated that immunization of C57BL/6 mice with DNA–histone complexes and MOG [18,19,40,41,42,43] accelerates EAE development. The acceleration is associated with specific changes in HSC differentiation profiles, an increase in lymphocyte proliferation, and apoptosis repression in different mice organs [18,19,39,40,41,42,43]. In EAE mice, these changes in parallel are associated with the production of auto-Abz splitting DNAs, RNAs, nucleotides, polysaccharides, proteins, and peptides [18,19,39,40,41,42,43]. After immunization of mice with MOG or DNA–histone complex, changes in HSC differentiation profiles occur several times, corresponding to the onset of the pathology by 7–8 days (the appearance of abzymes), a sharp exacerbation in the acute phase at 18–20 days (maximum activity of abzymes), followed by a slow transition to the remission stage and a decrease in the activity of abzymes in the hydrolysis of all above-mentioned substrates [18,19,39,40,41,42,43]. It was shown that during the spontaneous development of EAE, the levels of DNA, MBP, MOG, histones, and microRNA hydrolysis by Abzs increase gradually [18,19,39,40,41,42,43].

The most known alkaline phosphatase is an enzyme that removes a phosphate group (dephosphorylation) from many types of different molecules, including DNAs, RNAs, nucleotides, proteins, alkaloids, and other compounds [44]. This protein is a homodimeric enzyme with a molecular weight of 86 kDa. Each monomer contains five cysteine residues, two zinc atoms, and one magnesium atom, which are critical for its catalytic function [44]. This enzyme is most active at alkaline pH [45]. The enzyme occurs in various organisms, both eukaryotes and prokaryotes, with the same general function but in different structural forms appropriate to the environment in which they function. Alkaline phosphatase is revealed in the periplasmic space of *E. coli*. In humans, it occurs in many forms depending on its origin in the body—it plays an important role in liver metabolism and skeletal development. Due to its wide distribution in these areas, the phosphatase concentration in the bloodstream is used by diagnosticians as a biomarker to determine diagnoses such as hepatitis or osteomalacia [46]. Alkaline phosphatase affects inflammatory responses in patients with chronic kidney and is directly associated with anemia resistant to erythropoiesis stimulants [47]. Intestinal alkaline phosphatase regulates pH and ATP hydrolysis in the rat duodenum [48].In addition to alkaline, many other phosphatases have been described. Phosphatases are classified by substrate specificity and sequence homology in catalytic domains [49,50]. All phosphatases were divided into 104 distinct enzyme families [Alkaline phosphatase (EC 3.1.3.1)|Protein Target—PubChem. http://pubchem.ncbi.nlm.nih.gov›protein/EC:3.1.3.1; Create: 2022-08-31, Modify-2024-02], including acid phosphatase, alkaline phosphatase, endonuclease/exonuclease/phosphatase family, kinase, phosphatome, phosphotransferase, protein phosphatase. Despite their classification into over one hundred families, all phosphatases still catalyze the same general hydrolysis reaction [49,50]. Artificial Abzs against stable analogues of transition states of different chemical reactions are described [1,2,3,4,5,6]. Like artificial Abzs, natural abzymes are Abs against enzyme substrates acting as protein haptens, mimicking transition states of chemical reactions. Anti-idiotypic abzymes against catalytic centers of enzymes also possess catalytic activities [6]. Abzymes with some activities were not found in the blood of apparently healthy donors [51,52,53,54].

Antibodies with phosphatase activity were found earlier in the blood of rabbits immunized with DNA, RNA, DNase I, DNase II, and RNase A [18,19,55,56]. These data indicate that abzymes with phosphatase activity can be formed by immunizations of humans and animals with self-antigens containing phosphate groups (DNA, RNA, phosphorylated proteins, lipids, etc.). Synthesis of abzymes during immunization of animals with enzymes that cleave substrates containing phosphate groups such as phosphatases, RNases and DNases suggests the second way of producing abzymes with phosphatase activity as anti-idiotypic antibodies against active centers of some enzymes.

As stated above, in patients with AIDs, including MS and EAE, harmful abzymes with different catalytic functions are produced. In mammalian organisms, there are a lot of different molecules that contain phosphate groups. Considering this, a large number of various abzymes with varying phosphatase activities may be produced. Such abzymes may play a negative role in the pathogenesis of autoimmune diseases (including human MS and mice EAE) by reducing the concentration of blood and cell components, which must function in a phosphorylated state. Considering this, this work was the first to analyze the possibility of producing abzymes with phosphatase activity in EAE mice.

In addition, it was interesting to understand at what stages of EAE development the production of abzymes with phosphatase activity can occur. We used EAE-prone C57BL/6 mice, in which case it is possible to analyze changes in the phosphatase activity of IgGs during the onset, acute phase, and remission of this disease. Here, we investigated the phosphatase activity of IgG preparations from the blood plasma of C57BL/6 mice corresponding to spontaneous, MOG and DNA–histone complex, accelerating the development of EAE.

## 2. Results

### 2.1. Experimental Groups of Mice

The achievement of EAE leads to specific reorganization of the immune system of EAE-prone C57BL/6 mice associated with particular changes in the differentiation of mice HSCs [18,19,39,40,41,42]. All these specific defects of the immune system of mice lead to the increase in proteinuria (protein concentration in urea) and the generation of catalytically active antibodies hydrolyzing DNA, MBP, and MOG at early stages of EAE development [18,19,39,40,41,42].

As noted above, the formation of antibodies with phosphatase activity has been established so far only in the case of rabbits immunized with DNA, RNA, DNase I, DNase II, and RNase A [18,19,55,56]. It was interesting to see how the defects in the immune system of EAE mice can affect possible alterations in antibodies with phosphatase activity during different stages of EAE development. Further, it was interesting to compare the overtime patterns of changes in the relative phosphatase activity of Abs with those for abzymes hydrolyzing MBP, MOG, and DNA [18,19,39,40,41,42].

In the study of phosphatase activity, we used IgGs containing no canonical enzymes before and after immunization of C57BL/6 mice with MOG and DNA–histone [40,41,42]; these electrophoretically homogeneous preparations were obtained and described in [40,41,42,57,58]. To demonstrate the violation processes of C57BL/6 mice immune status, Supplementary data demonstrate overtime changes in a number of the bone marrow of mice CFU-E, DFU-E CFU-GM, and CFU-GEMM colony forming units (Supplementary Figure S1); the relative amount of lymphocytes in bone marrow, spleen, thymus, and lymph nodes (Supplementary Figure S2) for no immunized mice, as well as after their treatment with DNA–histone complex and MOG described in [40,41,42].

### 2.2. Phosphatase Activity Assay

The IgGs used here were obtained from the blood of mice previously and described in [57,58]. These IgG antibodies were active in the hydrolysis of DNA, MBP, and histones [40,41,42,57,58].

As stated above, mice develop spontaneous EAE, and disease is greatly accelerated after mice immunization with MOG or DNA–histone complex. To analyze changes in abzyme phosphatase activity, during spontaneous development of EAE from mice blood plasmas were purified IgGs corresponding to their life during 50, 90, 110, and 150 days after their birth.

Spont50: zero time, beginning of experiments at 50 days of mice age before their immunization,

Spont90: 3 mice-age mice (90 days) before their immunization,

Spont110: 110-day-old mice before their immunization, and

Spont150: development of EAE during 150 days without mice immunization.

After immunization of a 3-month-old mice with MOG, the maximum increase in the activity of DNase abzymes is observed after 20 days, while there are two stages of a strong rise in DNase activity—at 20 and 60 days after immunization—after mice treatment with DNA–histone complex. Taking this into account, antibody preparations were obtained corresponding to 20 and 60 days after immunization of mice with the DNA–histone complex.

MOG20: IgGs corresponding to 20 days after 3 mice-age mice immunization with MOG,

DNA20: IgGs corresponding to 20 days after 3 mice-age mice immunization with complex DNA–histone, and

DNA60: IgGs corresponding to 60 days after 3 mice-age mice immunization with DNA–histone complex.

**Figure 1:**
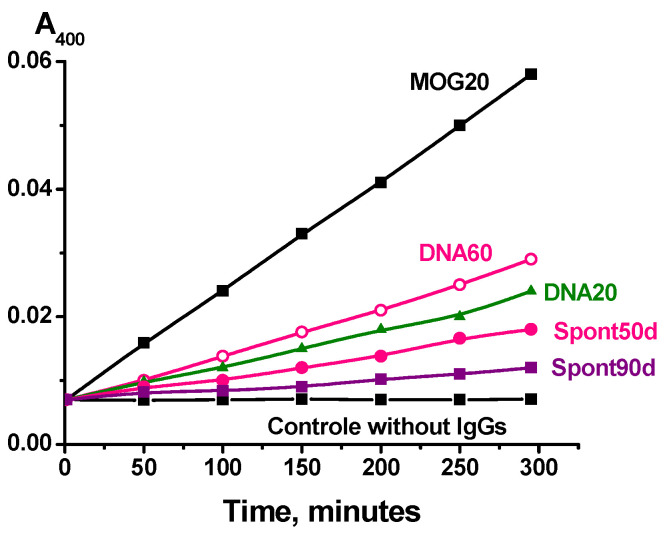
Typical examples of phosphatase activity determination using the increase in A_400_ of hydrolyzed *p*-nitrophenyl phosphate in the presence of five individual IgG preparations (0.1 mg/mL) at pH 8.8. All designations are given on Panel: IgGs corresponding to 20 days after immunization of mice with MOG (MOG20), or 20 (DNA20) and 60 (DNA60) days after mice treatment with DNA–histone complex, spont50d and spont90d preparations corresponding to IgGs isolated from the blood plasma of mice at 50 and 90 days after their birth before immunization with antigens. The error from two to three independent experiments in the initial rate determination did not exceed 8–10%.

The phosphatase activity of IgGs was analyzed using the change in the absorption (A_400_) during *p*-nitrophenyl phosphate hydrolysis. All IgG preparations hydrolyze *p*-nitrophenyl phosphate but at different rates. Figure 1 demonstrates five typical kinetic curves of phosphatase activity in the presence of several individual IgGs. Such kinetic curves were obtained for each IgG preparation.

**Figure 2:**
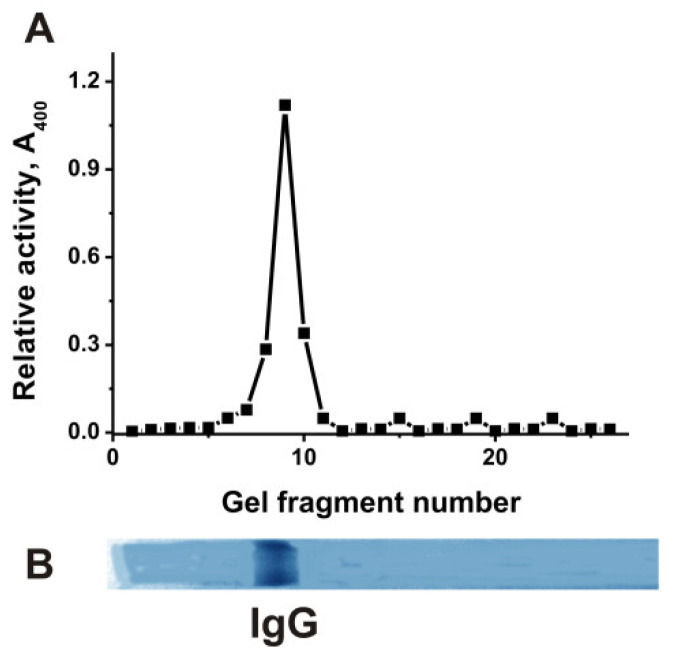
After SDS-PAGE of IgG_mix_ in 4–18% gradient gel, the gels were incubated using the special solution to remove SDS and IgG renaturation. The relative phosphatase activity was estimated using 17 µL extracts of 2.0–3.0 mm fragments of one longitudinal gel slice (**A**). The second longitudinal gel slice of IgG_mix_ corresponding to a mixture of 25 individual IgG preparation (19 µg) was stained with Coomassie blue (**B**). The error from two independent experiments in the initial rate determination did not exceed 8–10%.

Using the analysis of IgG activities in the hydrolysis of DNA, RNA, MOG, MBP, and histones, it was shown that all these enzymatic activities are the properties of IgGs [40,41,42,57,58]. To prove that the phosphatase activity belongs directly to mouse IgGs and not to any possible admixtures of canonical phosphatases, we used an equimolar mixture of 25 IgG preparations (IgG_mix_). After SDS-PAGE and removal of SDS, the gels were cut into small fragments (2–3 mm), and all possible components of these fragments were eluted from the gel species. The gel fragment eluates were used to analyze phosphatase activity (Figure 2).

The phosphatase activity was detected only in eluates corresponding to gel fragments containing 150 kDa IgG_mix_. Since SDS dissociates all complexes of proteins, the phosphatase activity was detected only in the gel fragment of intact IgG_mix_, together with the absence of any other protein bands and phosphatase activity in other gel fragments, providing direct evidence that IgG_mix_ possesses intrinsic phosphatase activity (Figure 2).

### 2.3. Optimal pH of Substrate Hydrolysis

As noted above, alkaline phosphatase has been found in the blood and all organs of mammals [44,45,46,47,48]. As shown by the example of abzymes with DNase and protease activities, in the blood of patients with AIDs and EAE mice, a pool of such abzymes usually contains specific fractions of antibodies capable of hydrolyzing DNA and proteins at several optimal pH values from 4 to 10 [18,19]. However, canonical enzymes with phosphatase activity can have several different optimal pH values [49,50]. Considering this, it was necessary to evaluate the optimal pH values of phosphatase-Abzs corresponding to different stages of spontaneous development of EAE and after mice treatment with MOG and DNA–histone complex.

**Figure 3:**
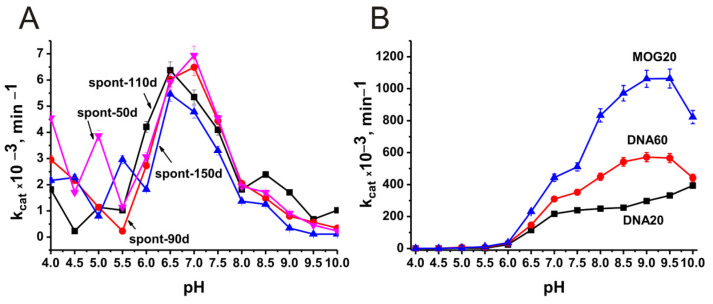
Dependence of the relative phosphatase activity of antibodies in the hydrolysis of *p*-nitrophenyl phosphate on different pH values of the reaction mixtures supplemented with 10 mM MgCl_2_ in the case of four antibody preparations corresponding to different stages of spontaneous development of EAE: 50 (spont-50d), 90 (spont-90d), 110 (spont-110d), and 150 (spont-150d) days after the birth of mice (**A**) and corresponding to the development of EAE by 3-month-old mice after their treatment with MOG (MOG20) or DNA–histone complex: DNA20 (20 days) and DNA60 (60 days) (**B**).

At the beginning, an analysis was made of the optimal pH values in the case of antibodies corresponding to different stages (50–150 days) of spontaneous development of EAE: 50 (spont-50d), 90 (spont-90d), 110 (spont-110d), and 150 (spont-150d) days after the birth of mice. Figure 3A shows the dependence of phosphatase activity on pH for these IgGs. For further analysis, we averaged data of apparent *k*_cat_ values corresponding to seven mice in each group. Figure 3B demonstrates the dependence of phosphatase activity on pH in the case of IgGs corresponding to mice immunized with MOG and DNA–histone complex.

As it is seen from Figure 3A, the pool of polyclonal IgGs corresponding to spontaneous development of EAE contains fractions of abzymes that hydrolyze *p*-nitrophenyl phosphate at very different pH values from 4 to 10. However, the main part of abzyme subfractions shows maximum activity at pH 6.5–7.0.

Immunization experiments were performed using 3-month-old mice (time zero, 90 days after birth of mice). An analysis was made of the optimal values of the pH of antibodies with phosphatase activity corresponding to the abzymes that are present in the blood of mice 20 days after mice immunization with MOG (MOG 20), 20 (DNA20) and 60 (DNA60) days after treatment of mice with DNA–histone complex. Figure 3B shows the dependence of the phosphatase activity of these IgGs on pH values.

Somewhat unexpectedly, immunization of mice with MOG and DNA histones complex greatly altered the formation of abzymes with phosphatase activity. Immunization of mice with antigens led to the production of lymphocytes producing antibodies that better hydrolyze the substrate at optimal pH values from 7.5 to 10.0 and especially 8.5–9.5 (Figure 3B). These antibodies, as well as abzymes corresponding to the spontaneous development of EAE, hydrolyzed *p*-nitrophenyl phosphate at pH 6.0 to 10.0. But to this zone, 6.5–7.0 pH values correspond only to the expressed shoulders of dependencies.

**Figure 4:**
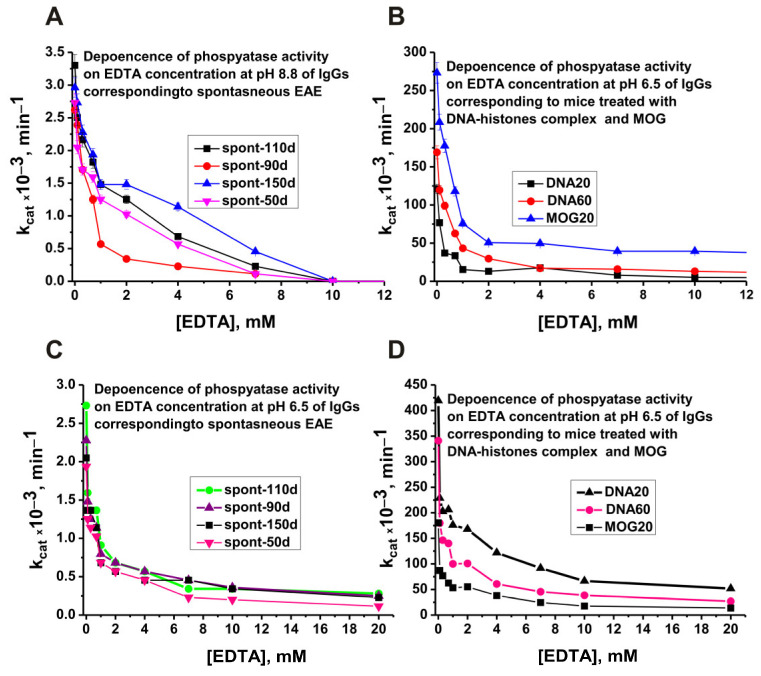
Dependences of the relative phosphatase activity at pH 8.8 and 6.5 on EDTA concentration of four preparations corresponding to different times of spontaneous development of EAE (50–150 days; **A**,**C**, respectively) and IgG preparations of 3-month-old mice after their immunization with MOG (MOG20) and DNA–histone complex (DNA20 and DNA60; **B**,**D**, respectively). All designations are given in the figure.

Basically, enzymes with phosphatase activity are activated by magnesium ions [46,47,48,49,50]. Therefore, all the experiments described above were carried out using 10 mM MgCl_2_ in the reaction mixtures. With this in mind, we first tested the effect of EDTA on antibody alkaline phosphatase activity at pH 8.8 and 6.5 (Figure 4).

As shown earlier, in the case of all studied abzymes that hydrolyze nucleic acids, proteins, polysaccharides, and other substrates, the antibody pool usually contains a large number of antibody subfractions differing in affinity for substrates, optimal pH values, hydrolysis rates, and other parameters [18,19]. As can be seen from Figure 4A,C, all IgGs that hydrolyze *p*-nitrophenyl phosphate at pH 8.8 and 6.5 are magnesium-dependent abzymes. The dependences of the phosphatase activity of the preparations MOG20, DNA20, and DNA60 on the EDTA concentration at pH 8.8 and 6.5 are given in Figure 4B,D. Interestingly, almost complete suppression of phosphatase activity for various IgG preparations is observed at different concentrations of EDTA. This indicates that different monoclonal antibodies with the phosphatase activity of IgGs differ in their affinity for magnesium ions.

**Figure 5:**
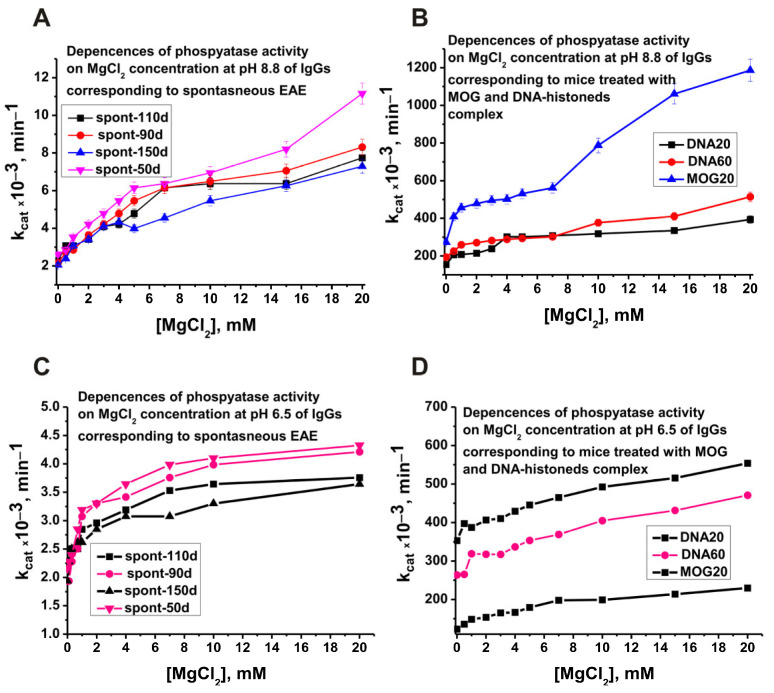
Dependences of the relative phosphatase activity at pH 8.8 and 6.5 on MgCl_2_ concentration of four preparations corresponding to different times of spontaneous development of EAE (**A**,**C**) and IgG preparations of 3-month-old mice after their immunization with MOG (MOG20) and DNA–histone complex (DNA20 and DNA60; **B**,**D**, respectively). All designations are given in the figure.

It was interesting to evaluate the optimal concentrations of magnesium ions that stimulate the activity of abzyme antibodies. Figure 5 demonstrates the dependencies of phosphatase activity on MgCl_2_ concentration of several preparations corresponding to spontaneous and antigen-induced development of EAE.

Interestingly, the dependences of phosphatase activity at pH 8.8 and 6.5 (Figure 5A,C) on MgCl_2_ concentration of antibodies corresponding to the spontaneous development of EAE and IgGs after immunization of mice with MOG and DNA–histone complex differ significantly (Figure 5B,D). The biphasic nature of the dependences on the concentration of magnesium ions is especially pronounced in the case of antibodies after immunization of mice with MOG (Figure 5B). In addition, the maximum increase in activity in the presence of magnesium ions is also observed for preparations after immunization of mice with MOG (Figure 5B). The whole set of data on the dependences of phosphatase activity on the concentration of magnesium ions of different preparations may indicate that being dependent on magnesium ions, various subfractions of monoclonal IgGs of antibodies pool differ in their affinity to these ions.

### 2.4. Changes in the Phosphatase Activity over Time during the Development of EAE

**Figure 6:**
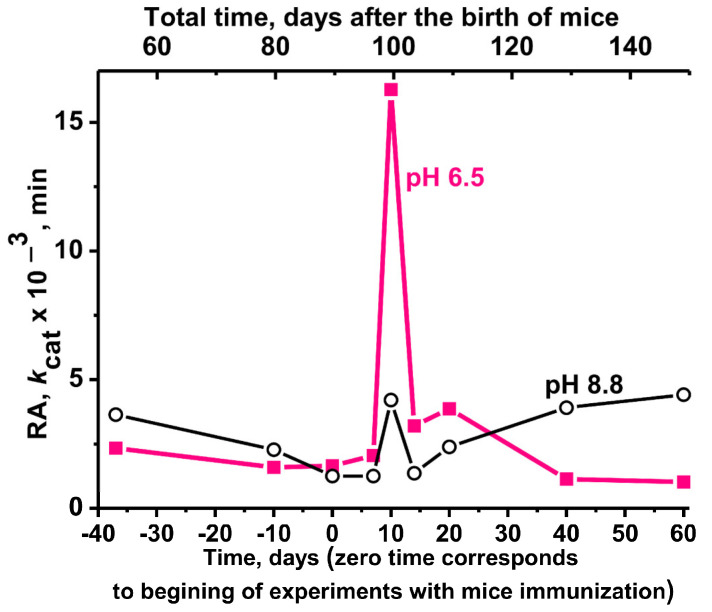
Changes in the average phosphatase activity of IgGs (7 mice in each group) over time during spontaneous development of EAE (50–150 days after birth, upper scale) at pH values of 6.5 and 8.8. The lower scale indicates the beginning of the experiments (zero time, see below) with immunization of mice with MOG and DNA–histone complex.

Taking into account the data on the maximum activity of different abzymes at pH 6.5–7.0 and 8.5–9.5 (Figure 3A,B), we first analyzed the change in the relative phosphatase activity of IgGs during the spontaneous development of EAE at two pH values, 6.5 and 8.8. Figure 6 shows the changes in average apparent *k*_cat_ values of IgGs during the spontaneous development of EAE measured using pH values of 6.5 and 8.8.

At 50 days after the birth of mice, the phosphatase activity of IgGs at pH 8.8 is approximately 1.6-fold higher than at pH 6.5. Quite unexpectedly, at 100 days after the birth of mice, a sharp but temporary, very strong increase in the phosphatase activity of antibodies is observed at pH 6.5 and 8.8 (Figure 6), wherein the activity of antibodies at pH 6.5 is 3.9-fold higher than at pH 8.8. Interestingly, with further spontaneous development of EAE from 20 to 150 days, a decrease in abzyme activity is observed at pH 6.5 but an increase at pH 8.8 (Figure 6).

**Figure 7:**
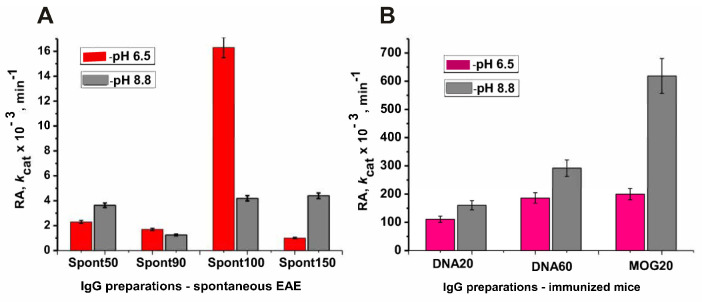
Changes in the average phosphatase activity of IgG preparations (7 mice in each group) over time during spontaneous development of EAE in 3-month-old mice from 50 to 150 days of life (**A**) and (zero time) mice immunization DNA–histone complex (20 and 60 days—DNA20 and DNA60) and MOG (20 days—MOG20) (**B**).

Immunization of mice with MOG and a DNA–histone complex leads to a strong increase in the phosphatase activity of antibodies-abzymes (Figure 7).

The lowest phosphatase activity of antibodies at pH values of 6.5 and 8.8 corresponds to 90 days of spontaneous development of EAE (zero time—the beginning of immunization of mice with immunogens) (Figure 7A); 20 (DNA20) and 60 (DNA60) days after immunization of mice with the DNA–histone complex (Figure 7B), phosphatase activity at pH 6.5 increased by 65.3 and 109.5 fold at pH 8.8.

A somewhat more significant increase in phosphatase activity is observed for DNA20 and DNA60 preparations at pH 8.8–128.4 and 233.6 fold, respectively. At pH 6.5, 20 days after immunization of mice with MOG, IG phosphatase activity was 117.6-fold higher than at time zero (Figure 7). However, the strongest increase in phosphatase activity, 494.7 fold, compared with zero time (90 days of life), occurred at pH 8.8 at 20 days after mice immunization with MOG.

Thus, in the process of spontaneous development of EAE, there is a specific change from 50 to 150 days after mice birth in the profile of stem cell differentiation, when differentiated lymphocytes appear, producing abzymes with different phosphatase activities. However, immunization of mice with DNA–histone complex and MOG, which accelerates the development of EAE, leads to some other changes in the differentiation profile of HSCs, leading to the appearance of lymphocytes producing much more active abzymes in the hydrolysis of *p*-nitrophenyl phosphate.

## 3. Discussion

An analysis of the catalytic activities of Abs-abzymes in the blood of patients with autoimmune and some viral diseases is of great interest since it allows obtaining utterly new knowledge that cannot be obtained using other immunology methods [18,19].

Theoretically, the immune systems of mammals could produce 10^6^ variants of antibodies against one specific antigen having very different properties [59]. As shown in a number of studies using monoclonal antibodies from the peripheral blood of SLE patients, the number of abzymes to the same antigen (DNA or MBP) with a variety of different activities can exceed 1000 (for review, see [18,19]). The pool of antibodies against each of these antigens contains monoclonal antibodies that differ in the rate of hydrolysis of these substrates, optimal pH values, isoelectric points, dependence or independence on various metal ions (Mg^2+^, Mn^2+^, Ca^2+^, etc.), substrate specificity, thermal stability, etc. [19]. In human blood, antibodies, and abzymes against the same antigen are extremely diverse [18,19]. The possibilities of affinity chromatography and ELISA in studying a possible diversity of Abs against different specific antigens in the blood plasma of conditionally healthy donors and patients with AIDs and viral diseases are very limited. An analysis of their significant difference in the totality of various properties allows only an analysis of their differences in very various enzymatic properties. Particularly striking in this regard are the data on the study of hydrolysis sites of five histones (H1-H4) by IgGs against these histones [18,19,57,58]. It has been shown that IgGs against each of the histones have cross-catalytic activity and hydrolyze each of these histones. It is especially interesting that Abs corresponding to various stages of spontaneous EAE development differ greatly in the number and type of sites of the hydrolysis of each of the five histones [57,58]. Moreover, the acceleration of the development of EAE after immunization of mice with MOG or DNA–histone leads to a change in specific sites of five histones hydrolysis [57,58]. In addition, even after immunization of mice with the same antigen, there are strong differences in the specific sites of each histone hydrolysis by IgGs corresponding to the onset, acute, and remission stages of EAE.

In this work, for the first time, we analyzed the phosphatase activity of IgG antibodies from the blood of EAE-prone C57BL/6 mice. As shown earlier, during the spontaneous development of EAE in C57BL/6 mice, a specific change in the differentiation profile of stem cells, leading to the production of abzymes hydrolyzing DNA, MBP, and histones, occurs at approximately 3 months (90 days) after their birth [40,41,42]. Therefore, it was strange that mice antibodies at 50 days have high phosphatase activity, which decreases markedly up to 90 days after their birth (Figure 6). However, at 110–120 days after the birth of mice, there is a sharp but temporary increase in the phosphatase activity of antibodies. It is possible that this time can probably correspond to the initial stage of accelerated spontaneous development of EAE.

It should be mentioned that treating mice with MOG and DNA–histone complex at time zero (3-month-old mice) leads to a significant increase in the activity of abzymes in the hydrolysis of DNA, MBP, and histones at 7–21 days after immunization [40,41,42]. The maximum activity of such abzymes is observed at 18–21 days (acute phase), with a subsequent decrease in activity after 25–30 days (remission stage). At the same time, at 20 days after mice immunization, DNase, MBP- and histones-hydrolyzing activities compared to zero time increased by 6–10 fold [40,41,42]. Phosphatase activity at 20 days (DNA20) and 60 days (DNA60) after immunization with complex DNA–histone rose much stronger: at pH 8.8–128.4 and 233.6 fold, respectively (Figure 7). At pH 6.5, at 20 days after mice treatment with MOG, IgGs were 117.6-fold more active than at time zero (Figure 7). However, the strongest increase in phosphatase activity, 494.7 fold, compared with zero time (90 days of life), occurred at pH 8.8 at 20 days after mice immunization with MOG. This is a very strong increase in the phosphatase activity of antibodies compared to abzymes that hydrolyze other above-mentioned substrates.

As mentioned above, monoclonal abzymes of patients with AIDs having the same enzymatic activity in pools of polyclonal antibodies can differ greatly in a large number of specific parameters. Moreover, at different stages of AIDs development, there are lymphocytes producing antibodies that hydrolyze DNA, MBP, and histones in entirely different ways [40,41,42]. A similar situation was revealed in the case of abzymes with phosphatase activity. For example, antibodies corresponding to the spontaneous development of EAE are able to hydrolyze *p*-nitrophenyl phosphate at pH values from 4 to 10, but still, the efficiency of substrate hydrolysis at pH 6.5–7.0 is much higher than at other pH values (Figure 3). Immunization of mice with MOG and DNA–histone complex leads to the appearance of lymphocytes producing antibodies that hydrolyze the substrate more efficiently at alkaline pH values of 8.5–9.5 (Figure 3). The dependences of the relative phosphatase activity on the concentration of magnesium ions for preparations corresponding to antibodies of spontaneous development of EAE and after treatment of mice with MOG and the DNA–histone complex also differ markedly (Figure 5). A pronounced biphasic curve of the dependence of IgGs-MOG20 activity on the concentration of Mg^2+^ ions indicates that the pool of IgGs after mice immunization with MOG contains antibodies with a lower and a higher affinity for magnesium ions (Figure 5). As noted above, the development of EAE in mice begins with a change in the differentiation profile of stem cells. Immunization of mice with MOG and a DNA–histone complex changes the differentiation profile and accelerates the development of EAE compared to its spontaneous development [40,41,42]. At first glance, immunization of mice with MOG could change the differentiation profile when lymphocytes produce antibodies and abzymes against MOG. However, as shown in a number of studies [18,19,40,41,42], the immune system’s response to MOG turns out to be extended, and lymphocytes abzymes are produced Abs that hydrolyze not only MOG but also DNA, MBP, five histones, and micro-RNA. In the case of treatment of mice with a DNA–histone complex, absolutely the same situation is observed [18,19,40,41,42].

As shown above, the relative phosphatase activity of antibodies rises with the spontaneous development of EAE and increases very strongly after immunization of mice with MOG and a DNA–histone complex. Interestingly, the increase in the relative activities of abzymes with the above-mentioned DNase, RNase, protease, and catalase activity in the acute phase of EAE (18–21 days after immunization), depending on the antigen varies from 5 to 40 fold [40,41,42,43]. At the same time, the phosphatase activity of antibodies after immunization of mice increases 65.3–497 fold. It turns out that immunization of EAE-prone mice with MOG and DNA–histone complex, stimulating a change in the differentiation profile of stem cells and accelerating the development of pathology, leads to an expanded formation of many different lymphocytes producing abzymes with at least several catalytic activities. As shown earlier in the example of mice not prone to the development of AIDs (CBA and BALB/c), their immunization with DNA–histone complex does not lead to a change in the profile of stem cell differentiation [39,40,41,42]. At the same time, the blood of such mice contains antibodies with significant DNase activity. However, their appearance is not associated with a violation of the differentiation profile of stem cells and is a consequence of the specific pre-differentiation of lymphocytes in different organs of mice [39,40,41,42]. In addition, immunization of these mice resulted in the appearance of DNA-hydrolyzing abzymes [39,40,41,42] but no other potential proteins, nucleotides, and polysaccharide substrates. Thus, the expansion of substrates for antibody hydrolysis is an exceptional feature of mice predisposed to the spontaneous development of AIDs. It should be assumed that the appearance of abzymes with phosphatase activity in C57BL/6 mice is associated with an extended effect of MOG and DNA–histone complexes on differentiating bone marrow stem cells.

Cells and blood contain a large number of different molecules of DNA, RNA, proteins, peptides, oligosaccharides, lipids, etc., some of which perform their biological functions by being phosphorylated. Against the many antigens containing phosphate groups, there may be a formation of abzymes with very different specific abzymes with phosphatase activity. The formation of abzymes with phosphatase activity can lead to dephosphorylation of these molecules and, as a result, eliminate their biological functions. Considering this, it can be assumed that abzymes with phosphatase activity may play a negative role in the pathogenesis of EAE.

## 4. Materials and Methods

### 4.1. Reagents

All chemicals, Protein G-Sepharose (17061801), and Superdex 200 HR 10/30 (17-5175-01) columns were from GE Healthcare Life Sciences (New York, NY, USA). MOG35–55 (2568/1) was from EZBiolab (Munich, Germany). Para-nitrophenyl phosphate was from Fisherscientific (Waltham, MA, USA, ICN10087801). These preparations were free from possible contaminants.

### 4.2. Experimental Animals

C57BL/6 mice, from 50 to 150 days after birth, were obtained from a mice special vivarium free of any pathogens at the Institute of Cytology and Genetics (ICG) [39,40,41,42,57,58]. All studies were performed according to the ICG’s bioethical committee, conforming to general principles of working with animals of Directive 86/609/CEE of the European Communities Council. The ICG’s bioethical committee supported our study. To analyze the changes in the phosphatase activity of IgGs during the spontaneous evolution of EAE, C57BL/6 mice from 50 to 150 days after mice birth were used. All immunization experiments were carried out using three-month-old mice (90 days of life; zero time) as described in [39,40,41,42,57,58].

### 4.3. Immunization of Mice

Here, we analyzed IgGs, which were used for the analysis of different specific parameters characterizing the evolution of EAE in C57BL/6 mice during the spontaneous development of disease and after mice treatment with MOG and DNA complex with five histones (H1-H4) [17,18,19,20,21]. At zero time (90 days or 3-month-old mice), EAE-prone mice were treated with MOG or DNA–histone complex according to [39,40,41,42,57,58]. To obtain IgG preparations, 0.7–1.0 mL of the blood was collected at 0–60 days after mice immunization by decapitation using standard approaches. The methods of mice immunization were published earlier [39,40,41,42,57,58], and they are described in more detail in Combined Supplementary data; Part 1, Immunization of mice.

### 4.4. IgG Purification

Electrophoretically homogeneous IgGs were isolated as in [39,40,41,42,57,58] by chromatography of mouse blood plasma components on Protein G-Sepharose and subsequent additional purification by FPLC gel filtration in harsh conditions (pH 2.6). All IgG samples were filtered using special 0.1 μm filters to protect IgGs from possible impurities. Aliquots of IgG solutions were kept at −42 °C before they were used in different experiments. SDS-PAGE of IgGs for assay of electrophoretic homogeneity was carried out using 4–15% gradient gels as in [39,40,41,42,57,58]. All possible proteins were visualized by gel silver staining. More detailed data concerning IgG purification are shown in Combined Supplementary data; Part 2. IgG purification.

To exclude possible artifacts due to hypothetically possible traces of contaminating phosphatases, IgG samples were separated by SDS-PAGE as in [39,40,41,42,57,58]. To restore the phosphatase activity after SDS-PAGE, SDS was removed by incubating gels for 5 h at 22 °C with Tris-HCl buffer (pH 7.5) and washed 14 times with this buffer. The longitudinal strips of gels were cut, and their small pieces (2.5–3.0 mm) were carefully pounded and placed in tubes with 50 μL of Tris-HCl buffer (pH 7.5) for 10 days at +4° C. The tubes were shaken using a mixer from time to time. Then, the gel particles were removed by centrifugation, and all supernatants were used to determine phosphatase activity. The parallel gel strips were used to reveal the IgG’s position by Coomassie blue staining. The phosphatase activity was found only in the protein band corresponding to intact 150 kDa IgGs, and there were no other protein peaks and phosphatase activity.

### 4.5. Phosphatase Activity Assay

The phosphatase activity of IgGs was analyzed according to [55,56]. Reaction mixtures (80 µL) contained 50 mM Tris-HCl pH 8.8 (or buffer with another pH), 0.2 mM *p*-nitrophenyl phosphate, MgCl_2_ at a concentration from 0 to 20 mM (mainly 10 mM), and IgG antibodies at a concentration of 0.02–0.1 mg/mL (more often 0.05 mg/mL). Depending on the preparation activity, the mixtures were incubated at 37 °C for 3–20 h (the optimal incubation time was selected for every preparation). All estimates were carried out within the linear regions of the time courses and IgG concentration curves. During incubation, the optical density of the reaction mixture was measured at 400 nm. All pH dependencies of phosphatase activity were analyzed using several different buffers (50 mM): citric acid-NaOH (4.0–5.0), MES-NaOH (pH 5.4–6.6), Tris-HCl (pH 6.0–8.8) and glycine-NaOH (pH 9.0–10.0). In some experiments, EDTA was added in reaction mixtures at a final concentration of 0–15 mM. To calculate *k*_cat_, the molar extinction coefficient of *p*-nitrophenol was used: ε = 18,300 M^−1^cm^−1^. [60]

### 4.6. Statistical Analysis

The results are given as the mean and standard deviation of 2–3 independent experiments for every IgG preparation, averaged for 7 mice in every group. The significant difference (*p*) between groups was calculated using the Mann–Whitney test.

## 5. Conclusions

EAE-prone C57BL/6 mice are characterized by the spontaneous and accelerated development of EAE after immunization with MOG and DNA–histone complex. EAE is associated with defects in the differentiation of bone marrow stem cells and production of antibodies-abzymes hydrolyzing MBP, MOG, DNA, RNA, and histones. Here, it was shown that different fractions of mouse polyclonal IgGs differ in terms of the optimal pH values of the reaction mixtures and are capable of hydrolyzing *p*-nitrophenyl phosphate at pH values from 4 to 10. However, the major fractions are antibodies that hydrolyze the substrate at pH 6.5–7.0 and 8.5–9.5. Specific defects of the immune status of mice during the spontaneous development of EAE lead to a 1.6–3.5-fold increase in the phosphatase activity of antibodies at pH 6.5 and 8.8 up. Immunization of mice with DNA–histone complex at different stages of EAE development (onset, acute phase, and remission) results in a 65.3- to 233.6-fold increase in phosphatase activity at these pH values. Maximal increases in phosphatase activity of 117.6 fold (pH 6.5) and 494.7 fold (pH 8.8) were observed at the acute phase of EAE after mice immunization with MOG. All data show that IgG phosphatase activity could be essential in EAE pathogenesis.

## Data Availability

All data are given in the article and Supplementary Materials.

## Supplementary Materials

The following are available online at: https://www.mdpi.com/article/10.3390/molecules29061382/s1; Combined supplementary data including Supplementary Figures S1–S4 and Supplementary methods (Part 1, Immunization of mice and Part 2, IgG purification). References [61,62,63] are cited in Supplementary Materials.

## Abbreviations

Abs, antibodies; auto-Abs, autoantibodies; BFU-E, erythroid burst-forming unit (early erythroid colonies); CFU-GM, granulocyte-macrophage colony-forming unit; CFU-E, erythroid burst-forming unit (late erythroid colonies); CFU-GEMM, granulocyte-erythroid-megacaryocyte-macrophage colony-forming unit; HSCs, hematopoietic stem cells; EAE, experimental autoimmune encephalomyelitis; SDS-PAGE, sodium dodecyl sulfate-polyacrylamide gel electrophoresis; SLE, systemic lupus erythematosus.
