# Supplementary material for: Autoantibodies-Abzymes with Phosphatase Activity in Experimental Autoimmune Encephalomyelitis Mice
Source: Molecules. 2024 Mar 20;29(6):1382. doi: 10.3390/molecules29061382 (PMC10975087; doi:10.3390/molecules29061382)
Supplement: Supplementary file 1 [file molecules-29-01382-s001.zip › molecules-2844258-supplementary.pdf]

Supplementary Figures

All Figures was taken and combined from the following articles:

1. Doronin, V. B., Parkhomenko, T.A., Korablev, A., Toporkova, L.B., Lopatnikova, J.A., et al. “Changes different parameters, lymphocyte proliferation and hematopoietic progenitor colony formation in EAE mice treated with myelin oligodendrocyte glycoprotein.” Journal of Cellular and Molecular Medicine, vol. 20, no. 1, pp. 81–94, 2016.
2. Aulova, K.S., Toporkova, L.B., Lopatnikova, J.A., Alshevskaya, A A., Sennikov, S.V., et al. “Changes in haematopoietic progenitor colony differentiation and proliferation and the production of different abzymes in EAE mice treated with DNA.”Journal of Cellular and Molecular Medicine, vol. 21, no. 12, pp. 3795–3809, 2017.
3. Aulova, K.S., Toporkova, L.B., Lopatnikova, J.A., Alshevskaya, A.A., Sedykh, S.E., et al. Changes in cell differentiation and proliferation lead to production of abzymes in EAE mice treated with DNA-Histone complexes. “Journal of Cellular and Molecular Medicine” vol. 22, pp. 5816-5832, 2018.
4. Tolmacheva, A.S.; Aulova, K.S.; Urusov, A.E., Orlovskaya, I.A.; Nevinsky, G.A. Increase in Autoantibodies-Abzymes with Peroxidase and Oxidoreductase Activities in Experimental Autoimmune Encephalomyelitis Mice during the Development of EAE Pathology. *Molecules* **2021**, *26*, 2077.
5. Mouse EAE models. *Overview and Model Selection Hooke Laboratories, Inc*; 2011-2013.
6. Andryushkova, A.S.; Kuznetsova, I.A.; Buneva, V.N.; et al. Formation of different abzymes in autoimmune-prone MRL-lpr/lpr mice is associated with changes in colony formation of haematopoetic progenitors. *J Cell Mol Med.* 2007; 11: 531-51.
7. Dubrovskaya, V.V.; Andryushkova, A.S.; Kuznetsova, I.A.; et al. DNA-hydrolyzing antibodies from sera of autoimmune-prone MRL/MpJ-lpr mice. *Biochemistry (Mosc)* 2003; 68:1081.

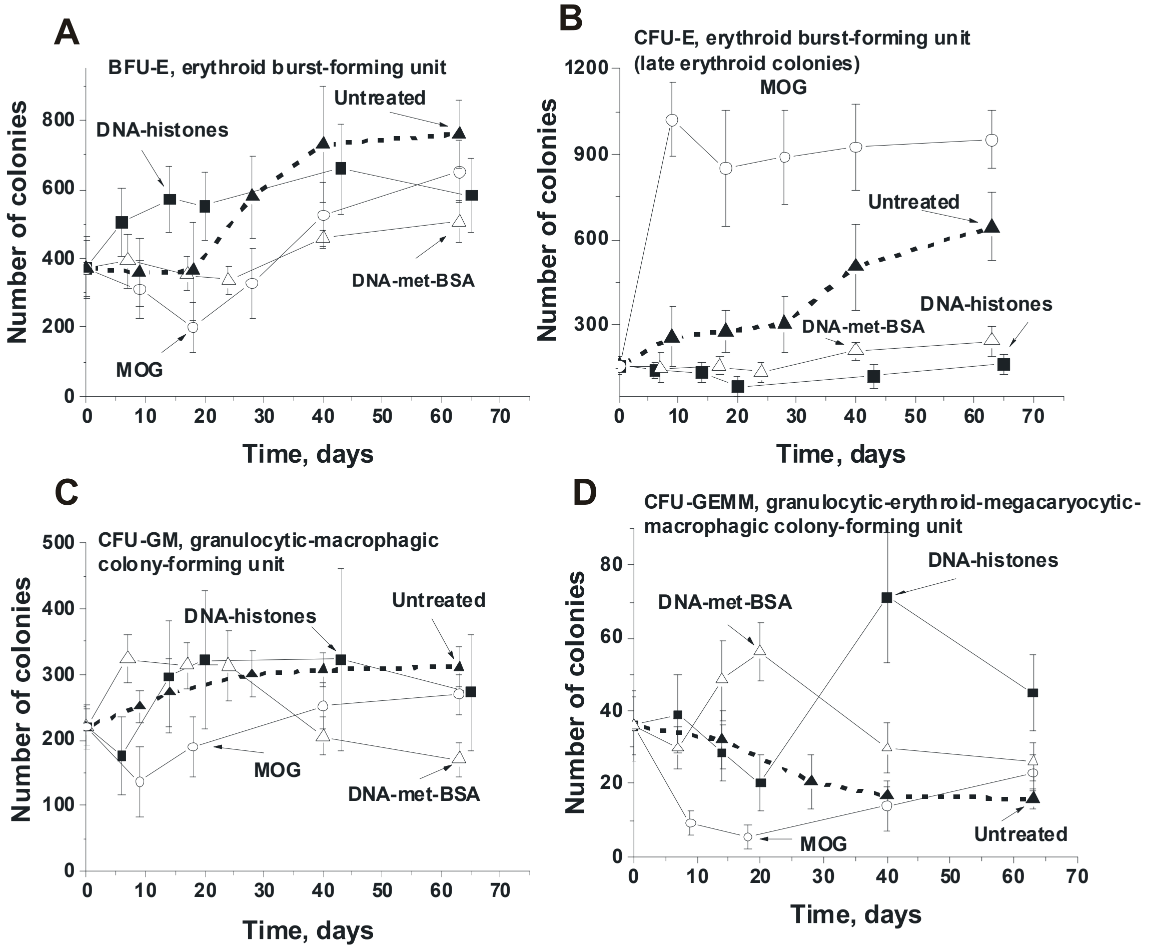

Supplementary Figure S1. Changes over time in a number of mice brain BFU-E (**A**), CFU-E (**B**), CFU-GM (**C**), and CFU-GEMM (**D**) forming colony units are shown for untreated mice, as well as after their treatment with DNA-histone, a complex of DNA with methylated BSA (DNA-met-BSA), and MOG [1-3]. Immunogens used are shown on Panels **A-D**.

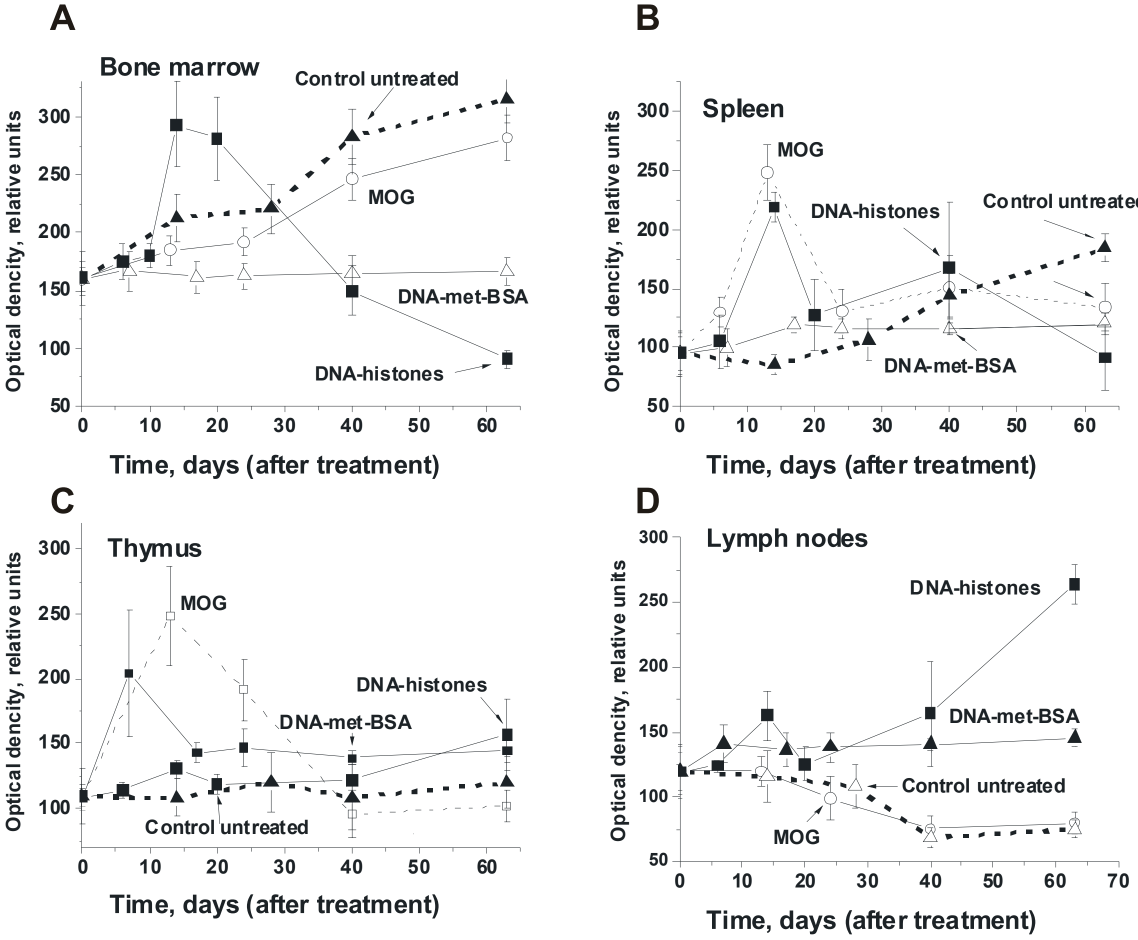

Supplementary Figure S2. The average over time changes in the optical density reflecting the relative amount of lymphocytes in bone marrow (**A**), spleen (**B**), thymus (**C**), and lymph nodes (**D**) are shown for untreated mice, as well as after their treatment with DNA-histone, a complex of DNA with methylated BSA (DNA-met-BSA), and MOG. Immunogens used are shown on Panels **A-D**. The error in the optical density estimation for each mouse for all groups (with seven mice per group) from three independent experiments did not exceed 7–10% [1-3].

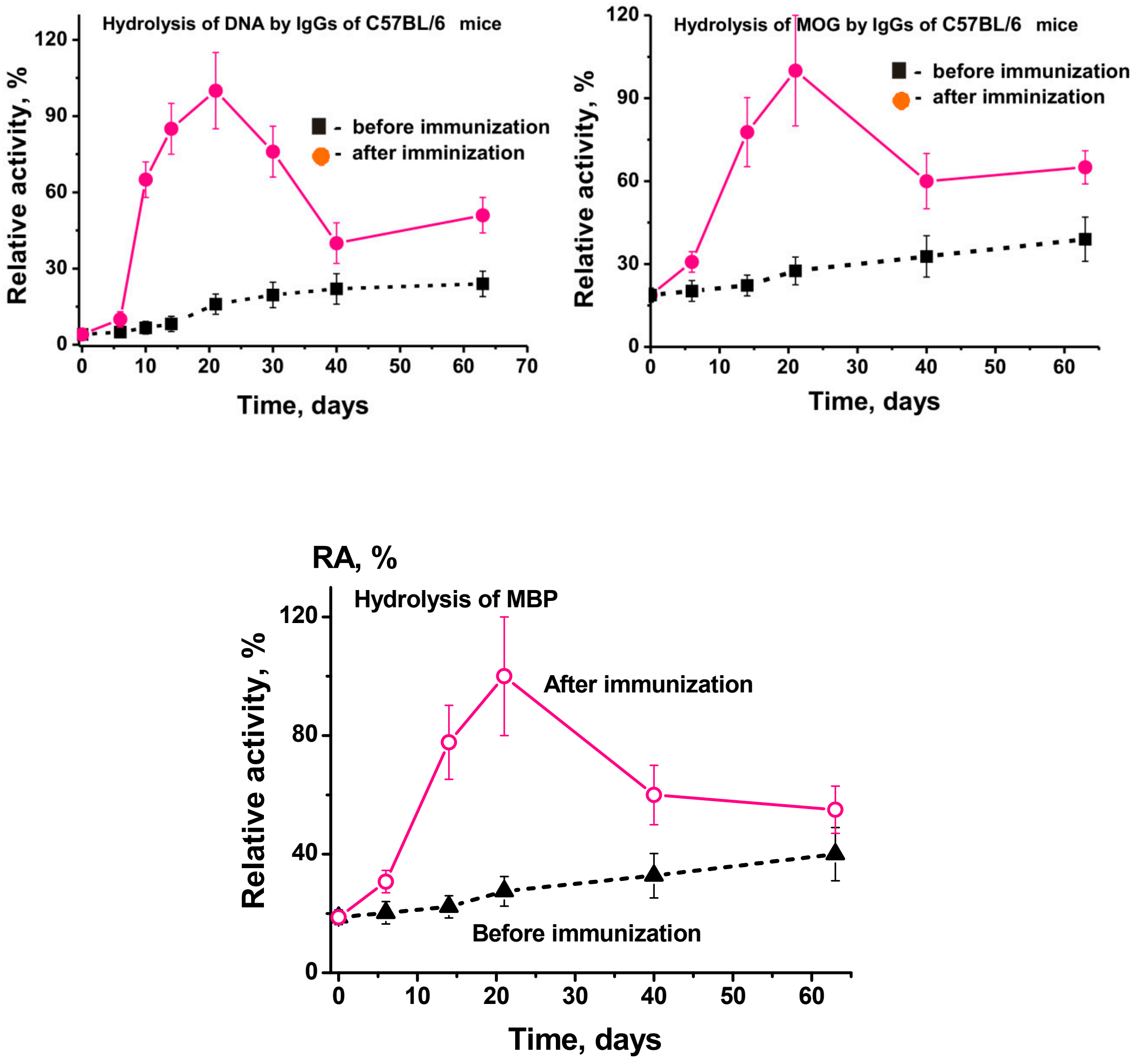

Supplementary Figure S3

Overtime changes of the average relative activities of IgGs of untreated and MOG-treated C57BL/6 mice (7 mice of each group) [1,2].

Supplementary Figure S4

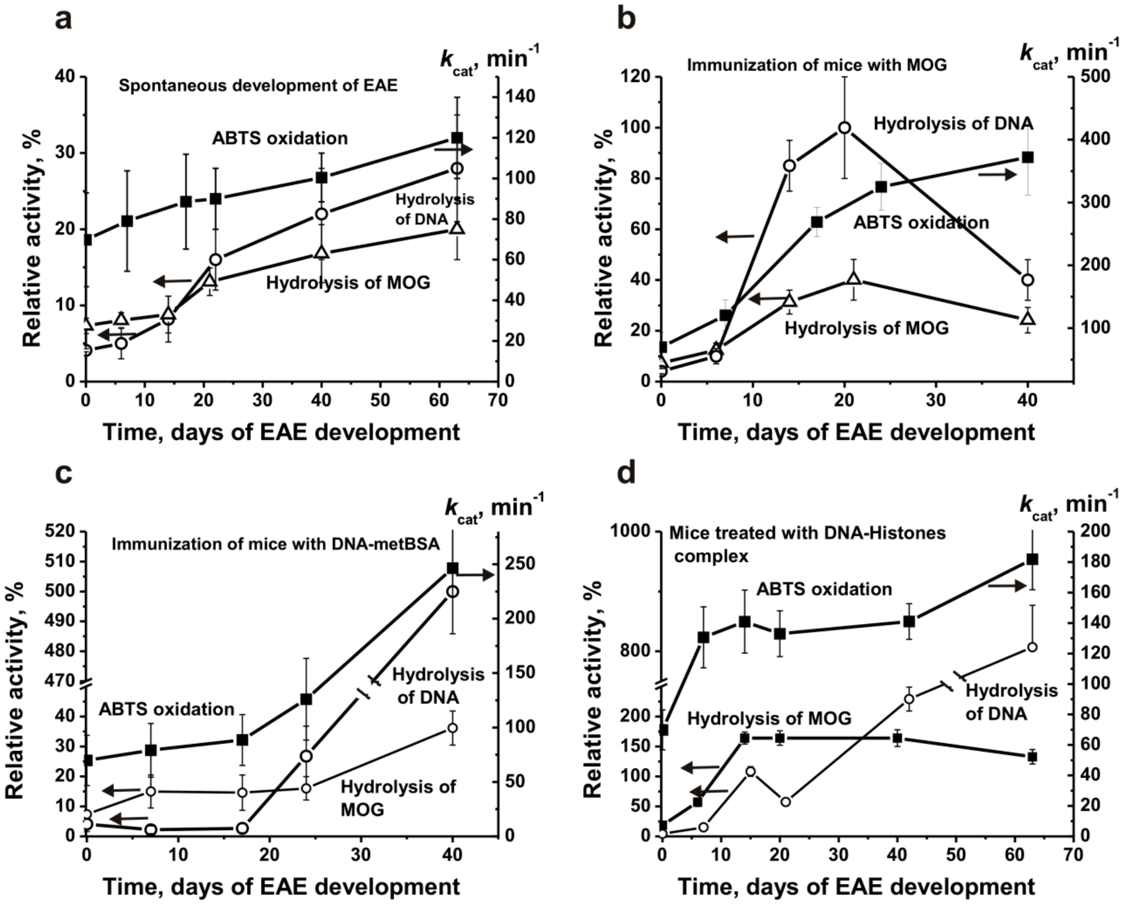

Supplementary Figure S4. Over time changes in the relative activity of IgGs against DNA and MOG in the hydrolysis of these substrates (**a-d**; left scales) as well as oxidation of ABTS (k_{cat}, min^{-1}; **a-d**; right scales). The mean values of the activities of IgGs from seven mice during spontaneous (**a**) as well as accelerated development of EAE after immunization of mice with MOG (**b**), DNA-metBSA (**c**), and DNA-histones (**d**) are given. All designations are marked in the Panels: the arrows in the Panels indicate to which Y axis the given curve belongs - to the right or to the left [4].

Supplementary methods

Part 1. Immunization of mice

Immunization of mice with MOG [1], the complex of DNA with methylated bovine serum albumin (DNA-metBSA) [2], and complex of DNA with five histones (H1, H2A, H2B, H3, and H4; DNA-histones) [3] was performed using Pertussis toxin (*Mycobacterium tuberculosis;* 0.4 µg) and Freund's adjuvant according to previously published protocol [5].

Polymeric thymus DNA was conjugated with methylated bovine serum albumin and dissolved in physiological solution as described previously [6,7]. The mixtures of MOG_{35-55} or the complex DNA-metBSA with Pertussis toxin and Freund's adjuvant and

obtaining their corresponding gels were carried out as described below for DNA-histones complex [1,2].

To prepare the complex DNA with histones a solution of 23.6 mg of a mixture of five histones (H1, H2A, H2B, H3, and H4; DNA-histones) in 11.8 ml of water was mixed with 23 mg of calf thymus DNA in 3 ml of water and 80 µl of 3 M NaOH (pH 10) was added; after complete dissolution, the mixture was titrated with 1 M hydrochloric acid to pH 7.0 and diluted with physiological solution containing 0.235 M NaCl to 18.8 ml. Then the mixture of 18.8 ml of antigen solution, 101.5 µg Pertussis toxin in 20 µl of water, and 18.8 ml complete Freund's adjuvant solution was used. This mixture was repeatedly passed through the syringe needle to form a homogeneous gel.

All gels for immunizing mice using MOG [1], the complex of DNA with methylated bovine serum albumin (DNA-metBSA) [2], and complex of DNA with five histones were obtained after mixing corresponding components with Pertussis toxin and complete Freund's adjuvant and passed through the syringe needle to form a homogeneous gel as described above for DNA-histones complex [3].

On day 1 (zero time), each C57BL/6 mouse was immunized by injection of 150-200 µl of gels containing 10 µg of MOG [1-3], DNA-metBSA (40 µg DNA) [2] the complex of polymeric DNA (94 µg) with histones (92 µg) [1-3] per mouse, as described below. The gels were injected subcutaneously (100-200 µl) into the clutches (50-100 µl). The second (after 2 days) immunization of each mouse has been performed in the same way using a 150 µl of a mixture of incomplete Freund's adjuvant containing 0.4 µg of Pertussis toxin. For different experiments including purification of antibodies and analysis of their enzymatic activity, 0.5-0.8 ml of blood was collected after decapitation using standard approaches.

Part 2. IgG purification

Electrophoretically and immunologically homogeneous mouse IgGs were obtained by sequential chromatography of the serum proteins on Protein G-Sepharose and following fast protein liquid chromatography (FPLC) gel filtration as described previously [1-3]. The serum protein (0.4–0.6 ml) was loaded onto a 1-ml protein G-Sepharose column equilibrated in buffer A (150 mM NaCl, 50 mM Tris-HCl, pH 7.5). The column was washed by buffer A to zero optical density (A_{280}). Proteins adsorbed non-specifically were eluted with the same buffer (15 ml) but containing 1% Triton X-100 and 0.3M NaCl and the column was washed with buffer A to zero optical density. The total IgGs fraction was eluted with 0.1 M glycine-HCl (pH 2.6), the

column fractions were collected to cooled tubes containing 50 ml of 0.5M Tris-HCl (pH 9.0), and finally each fraction was additionally neutralized with this buffer, concentrated for additional purification.

The purified IgG was incubated in acidic glycine-HCl buffer (pH 2.6) to disrupt non-covalent interactions and subjected to FPLC gel filtration on a Superdex 200 HR 10/30 column (Pfizer, New York,NY) using the BioCA workstation (Applied Biosystems, Foster City, CA) [1-3]. Abs were incubated for 20 min at 25° C in 0.1 M buffer (pH 2.6) containing 0.3 M NaCl and then subjected to the gel filtration on the column equilibrated in buffer A. The fractions of separated IgGs were collected and dialyzed against 20mM Tris-HCl (pH 7.5) containing 50 mM NaCl.

In order to protect Abs preparations from bacterial and viral contamination, they were filtered through Millex syringe-driven filter units (0.2 μm) and kept in sterilized tubes. Incubation of standard bacterial medium with stored Abs preparations did not lead to the formation of colonies.
